# Anterior Knee Pain and Excessive External Tibial Torsion in Female Patients: Rationale and Outcomes of Rotational Tibial Osteotomy

**DOI:** 10.3390/jcm15052015

**Published:** 2026-03-06

**Authors:** Vicente Sanchis-Alfonso, Jesus Castellano-Curado, Erik Montesinos-Berry, Santiago Ferrer-Piquer, Robert A. Teitge

**Affiliations:** 1 Department of Orthopaedic Surgery, Hospital Arnau de Vilanova, 46015 Valencia, Spain; 2 Department of Orthopaedic Surgery, Hospital Reina Sofía, 14004 Cordoba, Spain; 3 ArthroCentre Orthopaedics, Riaz & Clinique CIC Riviera, 1815 Montreux, Switzerland; 4 Department of Neurophysiology, Hospital Arnau de Vilanova, 46015 Valencia, Spain; safepiquer@gmail.com; 5 Department of Orthopaedic Surgery, Wayne State University, Detroit, MI 48202, USA

**Keywords:** anterior knee pain, patellofemoral pain, excessive external tibial torsion, rotational tibial osteotomy

## Abstract

Excessive external tibial torsion (ETT) is a recognized cause of anterior knee pain (AKP). In patients with excessive ETT, placing the foot forward during gait causes the knee joint to point inward, increasing the Q-angle and the lateral quadriceps vector. In appropriately selected cases, internal rotational tibial osteotomy is a reliable treatment option for symptomatic excessive ETT, yielding favorable outcomes with minimal complications. Nevertheless, no universally accepted torsion threshold exists to guide surgical decision-making, and evidence remains limited regarding the optimal anatomic level for performing the osteotomy.

## 1. Background

Most knee surgeons believe that the vast majority of anterior knee pain (AKP) cases do not require surgical treatment. They also believe that surgery often worsens symptoms and should therefore be avoided. However, studies show that only about 60% of AKP patients achieve satisfactory outcomes with appropriate conservative treatment [1]. This raises a key question: Why do 40% of AKP patients fail to improve with appropriate conservative treatment? The answer seems clear: we are missing certain causes of pain that are not amenable to conservative treatment but instead require surgical correction [2]. One such cause is torsional abnormality of the femur or tibia [2]. In a previous paper, we analyzed the treatment of AKP patients with pathological internal femoral torsion [3]. In this paper, we focus on pathological external tibial torsion (ETT) as a cause of AKP. Importantly, studies have found that 87% of patients in whom conservative treatment has failed have pathological ETT [4]. Still, not all of these patients are candidates for rotational tibial osteotomy.

Since the late 1970s, skeletal malalignment has been proposed as an etiology for AKP in young patients [5]. Skeletal malalignment is distinct from an abnormal Q-angle, increased TT-TG distance, or the position of the patella in the trochlea (i.e., increased displacement/subluxation or tilt); it refers instead to deviations of the limb in the transverse, coronal, or sagittal planes. Examples include femoral torsion, excessive ETT, and coronal deformities, all of which profoundly influence patellofemoral biomechanics. Among these, torsional abnormalities appear especially important [6,7,8].

In 1979, Stan James described miserable malalignment—increased femoral anteversion combined with increased ETT—as a cause of AKP [5]. In 1995, he reported seven such patients treated with internal rotational tibial osteotomy (IRTO) [9]. Even earlier, in 1990, Cooke et al. described proximal IRTO in seven AKP patients, highlighting the “squinting patella” as an unrecognized cause of symptoms [10]. Yet despite this, skeletal malalignment has been almost completely ignored in orthopedic surgery until relatively recently. In fact, very few publications mention torsional abnormality as a cause of AKP [11].

It is also interesting to note that in 1995, Flandry and Hughston [12] reported that the most common cause of failure in extensor mechanism realignment surgery was unrecognized, and therefore untreated, torsional abnormality. If torsional problems are not properly diagnosed, they remain untreated and surgical outcomes are poor [12]. In this regard, Stevens et al. have observed clinical improvements after rotational osteotomy in patients who had previously failed AKP surgery due to unrecognized torsional abnormalities [13]. Despite favorable outcomes [11], rotational tibial osteotomy remains underutilized and continues to generate controversy.

## 2. Why Is Excessive External Tibial Torsion a Problem? Why Do We Perform a Rotational Tibial Osteotomy?

**Figure 1:**
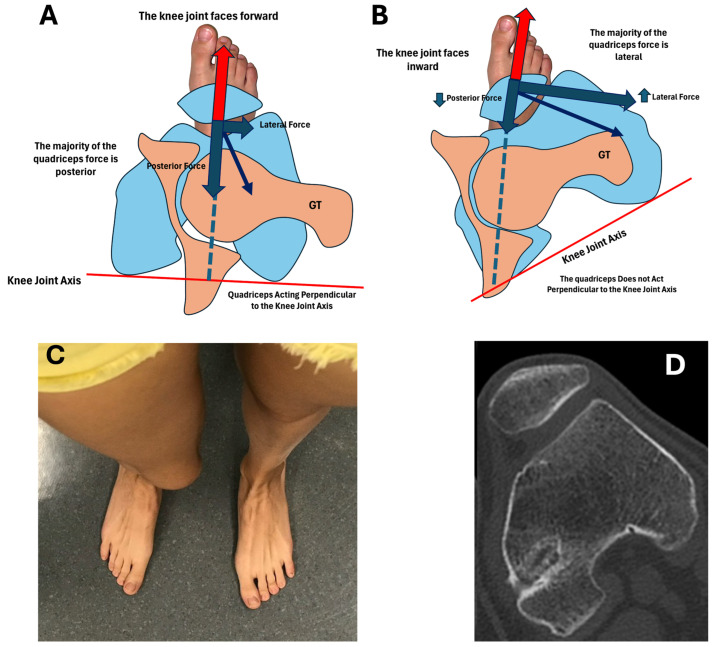
(**A**) Normal female with femoral anteversion (FAV) of 13° and ETT of 27°. (**B**) Female with 43° of ETT. To keep the foot progression angle normal, the knee joint axis (red line) points inward causing increased strain on the knee. The hip is markedly internally rotated with the greater trochanter (GT) oriented anteriorly. (**C**) Female with 43° of right-side ETT. (**D**) Computed tomography scan of the patient’s right knee shown in image C. The knee joint faces inward. This patient has a FAV of 15° and an ETT of 43°. (**C**) Republished from reference [15]. Published by Elsevier Inc. on behalf of International Society of Arthroscopy, Knee Surgery and Orthopedic Sports Medicine.

Many years ago, in 1948, Levens et al. [14] demonstrated that during normal gait, the knee joint axis moves straight forward in the direction of body movement, with only minimal (<10°) inward or outward deviation (Figure 1). However, in cases of excessive ETT, when the foot is placed forward during gait, the knee joint points inward [15] (Figure 1), which increases the Q-angle and thereby the lateral quadriceps vector [6]. When the knee joint axis is rotated out of its normal alignment relative to the body’s line of progression, the patellofemoral joint (PFJ) loading vectors are directed obliquely, creating abnormal shear forces [6].

Inward-pointing knees increase the lateral pull of the quadriceps while the posterior force decreases [6]. Reduced posterior force predisposes to knee collapse during flexion, forcing the quadriceps to contract more strongly to maintain stability. This amplifies both lateral and posterior components of the force vector, increasing strain on the medial patellofemoral ligament (MPFL) and medial meniscopatellar ligament while also altering patellar contact pressures, elevating stress on the lateral facet and reducing it medially [6]. This imbalance increases stress on PFJ ligaments and distorts force distribution across the PFJ articular surface.

In summary, maltorsion distorts the magnitude and direction of PFJ forces, and if these exceed biological tolerance, instability or arthrosis may follow. Many surgeons focus on damaged structures (cartilage, ligaments) rather than on the abnormal forces causing that damage [3]. Treating the injured structure without correcting the underlying force abnormality often leads to failure [3]. Although rotational osteotomy does not alter trochlear geometry or PFJ ligament integrity, it changes the force direction and magnitude, addressing the underlying issue.

**Figure 2:**
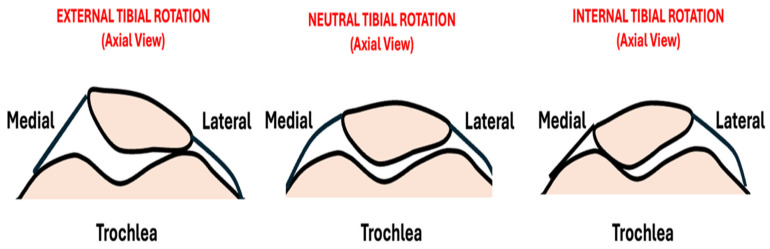
Drawings demonstrating the effect of tibial rotation on the PFJ. External tibial rotation increases the contact on the lateral side while internal tibial rotation increases the contact on the medial side.

The cadaveric work by Lee et al. [16,17] supports the importance of ETT (Figure 2). Their study showed that as little as 15° of external tibial rotation beyond the neutral position produced a significant increase in PFJ contact pressure, precisely localized on the lateral facet of the patella. While both internal and external tibial rotation increased PFJ pressures, external rotation produced far greater increases across all flexion angles. They also noted a decreased total PFJ contact area at 0°, 30°, and 60° of knee flexion.

**Figure 3:**
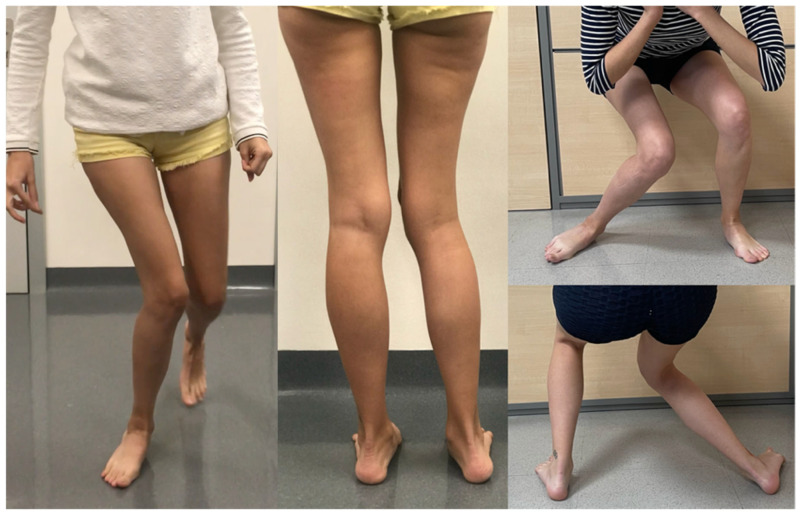
Excessive ETT tends to produce excessive internal tibial torque, which forces the subtalar joint into pronation and makes the foot appear pathologic. In addition, excessive ETT thrusts the knee joint axis medially, creating valgus forces at the knee. Republished from reference [15]. Published by Elsevier Inc. on behalf of the International Society of Arthroscopy, Knee Surgery and Orthopedic Sports Medicine.

Finally, excessive ETT tends to produce excessive internal tibial torque, which forces the subtalar joint into pronation and makes the foot appear pathologic (Figure 3). The less internal torque the more the foot arch appears normal. Patients with lateral patellar instability often forcibly internally rotate the tibia to reduce lateral patellar force. Likewise, patients landing from a jump often position the ankle joint axis in the coronal plane; in those with excessive ETT, this thrusts the knee joint axis medially, creating valgus forces at the knee. These biomechanical patterns offer important clues as to how excessive ETT contributes to abnormal gait (scissoring gait) in some AKP patients with pathological ETT.

## 3. Surgery

### 3.1. What Is the Threshold for Surgical Indication?

A substantial body of literature has sought to establish reference values for ETT in young, asymptomatic populations. Drawing on anthropometric measurements, cadaveric studies, and imaging-based assessments, the normal range of ETT in individuals of European descent is generally reported to lie between 24° and 30° [18]. At present, however, no universally accepted threshold clearly distinguishes physiological from pathological ETT.

In the majority of published surgical series, a value of 30° has been commonly adopted as a practical threshold for considering surgical correction, as osteotomy at or beyond this level has been associated with improvements in both clinical outcomes and rotational measurements [19,20,21,22]. In contrast, other authors recommend reserving surgical treatment for cases in which ETT exceeds 40° [23]. The patient with the lowest degree of torsion operated on in our series with an excellent result had 36° of ETT. For this reason, our current cutoff value was set above 35°.

Importantly, these numerical values should not be interpreted as standalone indications for surgery. In our practice, operative treatment is considered only in patients presenting with severe, functionally limiting AKP that persists despite adequate conservative management. To date, there is no robust evidence supporting the use of specific torsional angle cutoffs as definitive criteria for surgical indication [24].

**Figure 4:**
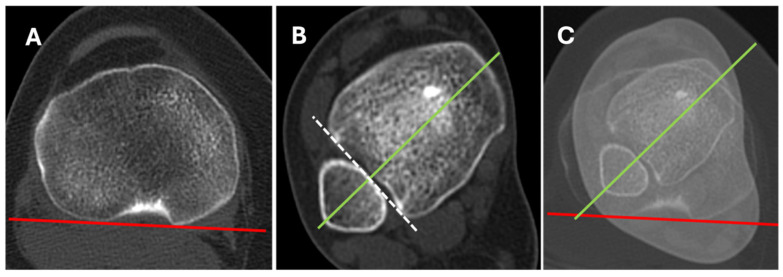
Tibial torsion measurement. (**A**). Axial CT image of the tibial plateau. The tangent to the posterior border of the tibial plateau forms the proximal line of reference (red line). (**B**) Axial CT image of the distal plafond and ankle malleoli. The line joining the most protruding part of the lateral and medial malleolus (green line), perpendicular to the fibular notch of the tibia (white dotted line) is the distal line of reference (green line). (**C**). Composite CT image of both tibial plateau and malleoli. Tibial torsion is measured as the angle between the proximal and distal reference lines. Republished from reference [26] © 2025 The Author(s). Journal of Experimental Orthopaedics published by John Wiley & Sons Ltd. on behalf of European Society of Sports Traumatology, Knee Surgery and Arthroscopy.

Based on the experience of the first author and his interpretation of the literature, disabling AKP recalcitrant to at least 6 months of adequate conservative treatment with ETT > 35°, measured with the Jend method [25] (Figure 4), is his current threshold for IRTO [24]. IRTO is contraindicated in the following situations: (1) AKP associated with ETT ≤ 35°; (2) tibial torsion deformity without clinical symptoms.; and (3) correction for cosmetic reasons [24].

### 3.2. What Is the Ideal Level for the Osteotomy?

One of the most controversial aspects of this surgery is determining the optimal level for performing the osteotomy. Most authors argue that the osteotomy should be supratuberositary because it can correct the Q-angle or the TT-TG distance when these are excessive [9,27,28,29,30,31,32,33]. Only a few maintain that it should be infratuberositary [6,13,34,35]. However, an alternative that is becoming increasingly popular for correcting ETT is supramalleolar osteotomy [36]. Supramalleolar osteotomy may be safer for the average surgeon as it avoids possible complications such as peroneal nerve palsy, compartment syndrome, and muscle damage. It is a reasonably good alternative but has some drawbacks. (1) Some patients complain of an exuberant callus that can be felt and seen. (2) The only satisfactory location for a fixation plate is medial; there is no space laterally due to the tibiofibular syndesmosis, and there is very little soft tissue to cover a medial plate. (3) The plate will probably not match the exact contour of the bone and be separated from the bone which may stretch the skin. (4) If we perform a 25° tibial rotation, the fibula fragments will separate so much that they will lose contact with each other. This will cause pain and a very prominent callus. Therefore, a long oblique osteotomy should be performed to increase the surface area and enable bone contact. (5) All tendons crossing the ankle must undergo a fairly abrupt change of direction with rotation, and we do not know what functional repercussions this will have. (6) Most of the medial plates must be removed because they are subcutaneous, which is not a complication but just another consideration. (7) In addition, the osteotomy must be proximal enough to have sufficient bone grip on the distal fragment.

In principle, a corrective osteotomy should be carried out as close as possible to the site of the deformity. However, the precise level at which abnormal tibial torsion originates cannot be reliably identified and may involve the entire length of the tibia. In female patients with AKP, it has been demonstrated that the infratuberositary component of pathological ETT becomes more pronounced as the severity of torsion increases [26]. This observation supports the infratuberositary region as a suitable level for surgical correction.

Nevertheless, determination of the optimal osteotomy level should not be based solely on the presumed origin of the torsional deformity, but also on the position of the tibial tuberosity (TT). When the TT is lateralized, a supratuberositary osteotomy may be justified. In contrast, if the TT is not lateralized, performing a supratuberositary osteotomy would lead to excessive medialization of the TT, resulting in increased medial femorotibial and PFJ loading. Such altered load distribution may contribute to the development of osteoarthritis over time [37,38].

In cases where the TT is not lateralized, an infratuberositary osteotomy is therefore preferred in order to avoid unintended medialization of the TT. Previous investigations have shown that in young women with AKP and pathological ETT, the TT is typically not lateralized [26]. Consistent with other clinical studies [39,40,41], our results further confirm that increased ETT is not associated with lateral displacement of the TT. These findings support the use of infratuberositary osteotomy as the treatment of choice for correction of severe ETT in female patients with AKP in the majority of cases.

### 3.3. What Should Be Done When There Is an Increased TT-TG Distance?

Patients with increased TT–TG distance and pathological ETT may appear to require supratuberositary osteotomy. However, several studies have shown that the TT-TG distance can be overestimated due to various factors, such as patient anatomy, position, and knee rotation [42,43,44,45]. Studies show that TT–TG distance is highly sensitive to rotational alignment, with cadaver and computational models demonstrating a change of about 0.5–0.6 mm for every degree of femorotibial knee rotation [46,47]. In patients with patellar instability, differences in TT–TG are driven more by tibio-femoral (knee) rotation than tubercle translation, and 73% of elevated TT–TG cases involve a rotational component—either alone or combined—compared with only 8% due solely to translation [48].

Recent evidence indicates that an increased knee rotation can overestimate the TT–TG distance by up to 0.84 mm per degree, a finding consistently demonstrated in CT-based studies of female patients with AKP [49]. After correcting for rotation, the TT–TG value typically falls below the 20 mm threshold in most cases, making it advisable to apply this correction particularly in patients whose knee rotation exceeds population averages (approximately 8–9° in most studies). Based on all of this, we believe that the best way to assess the true lateralization of the TT, and therefore indicate a supratuberositary osteotomy, is using alternative parameters such as the Tibial Tubercle Lateralisation (TTL) [50], or, if relying on TT–TG, ensuring that a Rotational-Corrected TT–TG is used [49].

### 3.4. What Should Be Done When There Is a Varus Associated with ETT?

In most of the cases the varus associated with ETT is not real. It is a reflection of the tibial torsion. Therefore, we must check if there is a neutral coronal plane alignment after rotation before fixation. We must remember that the normal mechanical axis is actually near the medial tibial spine, not in the center of the knee joint. To evaluate the mechanical axis an alignment rod is used from the center of the femoral head to the center of the talus to make sure the mechanical axis falls near the medial tibial spine. For this mechanical axis check, the patella must point straight forward, and it must be in the middle of the distal femur on the anteroposterior image. When the patella is centered in this way, the flexion–extension axis of the knee usually lies in the frontal (coronal) plane, allowing reliable evaluation of the mechanical axis.

### 3.5. Is Peroneal Nerve Release Needed? How to Anticipate Peroneal Nerve Palsy: Peroneal Nerve Release vs. Intraoperative Monitoring

IRTO carries a risk of excessive tension on the peroneal nerve. In earlier stages of our experience, a prophylactic peroneal nerve release was performed when the planned rotational correction exceeded 20°. At present, the first author routinely employs intraoperative peroneal nerve monitoring, with particular attention during internal rotation of the tibia.

One effective strategy to reduce the risk of peroneal nerve palsy is continuous intraoperative nerve monitoring. Since this approach was incorporated into our standard practice, this complication has not been observed again [24]. Real-time monitoring enables prompt identification of nerve irritation or excessive strain, allowing the surgeon to immediately interrupt the internal rotation maneuver of the distal tibial fragment without the need to awaken the patient. When relevant nerve irritation is detected before completion of the planned correction, the rotation is partially reversed until electrophysiological signals normalize.

Based on our experience, minor undercorrection does not appear to compromise clinical outcomes. Consequently, since adopting routine intraoperative peroneal nerve monitoring, we have discontinued systematic peroneal nerve release, even in cases requiring correction greater than 20°.

### 3.6. How Is Intraoperative Neurophysiological Monitoring Performed?

**Figure 5:**
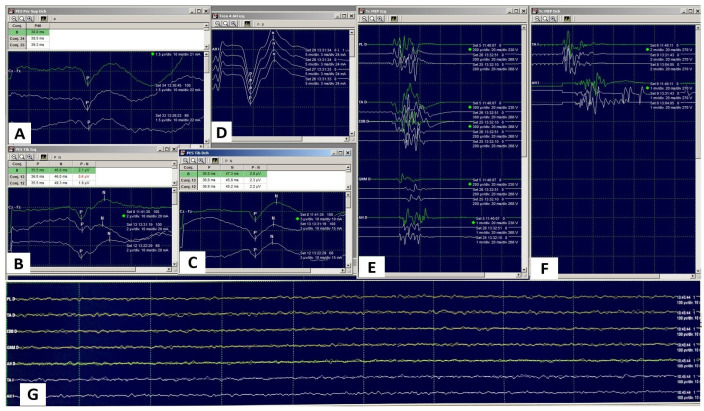
Right peroneal nerve (**A**) and Bilateral tibial posterior nerve (**B**,**C**) somatosensory evoked potentials, Right limb (study lower limb) (**E**) and Left limb (control lower limb) (**F**) transcranial motor evoked potentials. Free-running electromyography (**G**) records spontaneous muscle electrical activity and ongoing muscle electrical activity that happens caused by irritation, compression or stretching to a motor nerve (no stimulation needed). ToF (**D**) assesses the depth of neuromuscular blockade with four muscular responses.

Intraoperative neurophysiological monitoring is performed to enable early identification of potential neurological alterations. The common peroneal nerve may be injured during fibular head osteotomy and, especially, during the internal tibial rotation maneuver due to a stretch mechanism. Multimodal monitoring with evoked potentials and free-running electromyography is used in our intraoperative protocol (Figure 5).

**Figure 6:**
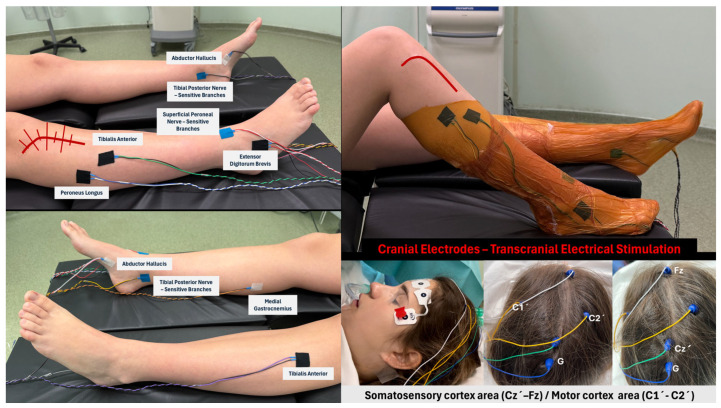
Location of needle electrodes for monitoring common peroneal nerve. Right limb (study lower limb), Left limb (control lower limb). Cranial electrodes are placed according to the international 10–20 system. The sensory signal is recorded from the somatosensory cortex corresponding to the knee region (Cz’-Fz). Additionally, two electrodes are placed at C1′ and C2′ to stimulate the motor cortex via transcranial electrical stimulation, triggering measurable motor responses in the target muscles (tibialis anterior, peroneus longus, and extensor digitorum brevis). Cranial electrodes image republished from reference [24] © 2025 The Authors. Published by Elsevier Inc. on behalf of International Society of Arthroscopy, Knee Surgery and Orthopedic Sports Medicine.

Somatosensory evoked potentials (SSEPs) are obtained by stimulating a peripheral nerve using a pair of subdermal needle electrodes (cathode–anode) placed along its superficial anatomical course in the limb (Figure 6), with cortical responses recorded from the somatosensory cortex using scalp electrodes positioned according to the international 10–20 system (Cz′–Fz) (Figure 6). The superficial peroneal branch (nerve at risk) is stimulated at the anterior border of the ankle (intermalleolar midline), and the posterior tibial nerve (control nerve) is stimulated at the medial retromalleolar region of the ankle.

Transcranial motor evoked potentials (TcMEPs) are elicited by electrical transcranial stimulation of the motor cortex (C1–C2) (Figure 6) and recorded from muscles innervated by the neural structures under evaluation (peroneus longus, tibialis anterior, and extensor digitorum brevis for the monitored peroneal nerve; medial gastrocnemius and abductor hallucis for the posterior tibial nerve used as control) (Figure 6).

To ensure the reliability of motor recordings, the depth of neuromuscular blockade is assessed using the train-of-four (ToF) technique. A ToF count of 0 reflects deep neuromuscular blockade and is associated with less reliable motor evoked potential recordings. In contrast, a ToF count of 4 (equivalent to a ToF ratio of 100%), defined by the presence of four full muscular responses to stimulation, indicates the absence of neuromuscular blockade and ensures fully reliable transcranial motor evoked potential responses. In addition, SSEPs and TcMEPs from the contralateral posterior tibial nerve and TcMEPs from the contralateral common peroneal nerve are obtained, allowing discrimination of response variations not attributable to surgical maneuvers on the operated limb (typically related to changes in anesthetic management or blood pressure drops), along with ToF monitoring of the posterior tibial nerve.

A total intravenous anesthesia (TIVA) approach is recommended, avoiding bolus administration and halogenated agents. Continuous evaluation of evoked potential latencies and amplitudes must not show significant changes during the critical stages of surgery.

### 3.7. Is Fibular Osteotomy Needed?

In earlier phases of our practice, a fibular osteotomy was routinely performed when rotational correction exceeded 20°. Currently, this step is reserved for situations in which mechanical resistance prevents smooth execution of the planned tibial rotation. When the fibula remains intact, rotational forces may be transmitted across the proximal and distal tibiofibular joints and potentially through the fibular shaft itself. This mechanism has been proposed as a possible explanation for postoperative pain at these joints following IRTO performed without a fibular osteotomy. For this reason, a fibular osteotomy should be considered when substantial tibial rotational correction is anticipated.

Nevertheless, according to the clinical experience of the first author, no pain at the proximal or distal tibiofibular joints has been observed when a fibular osteotomy was omitted, even in cases involving corrections of 20° or more, although such large corrections were uncommon in our series.

When a fibular osteotomy is required, we prefer a proximal long oblique osteotomy without bone block excision. This configuration allows minimal displacement, is associated with less postoperative pain, and demonstrates faster healing. In contrast, a straight transverse fibular osteotomy tends to be more painful and shows delayed union. We do not perform release of the proximal tibiofibular joint, as this maneuver carries a risk of postoperative instability.

During fibular osteotomy at the level of the fibular neck, the peroneal nerve is particularly vulnerable. To minimize this risk, the nerve is protected using two hallux retractors positioned around the fibular neck while the osteotomy is performed with a small oscillating saw. In our view, a substantial proportion of peroneal nerve palsies are likely related to displacement at the fibular osteotomy site.

### 3.8. Outcomes

Only a limited number of studies have evaluated the outcomes of IRTO for the management of AKP in otherwise healthy young patients [9,10,21,22,24,29]. Overall, these reports describe favorable clinical results with a low incidence of complications. In a recent systematic review, the pooled rate of persistent postoperative AKP was 7.6% (95% CI: 0.7–18.8%) [51]. The results of our series are consistent with these findings, demonstrating marked symptom relief and functional improvement in a carefully selected patient population [24]. In the personal series of the first author, 15.38% of the operated patients underwent bilateral surgery.

The present series includes twenty-six infratuberositary IRTOs performed exclusively in female patients, with a mean age of 22 years and a mean follow-up of 5 years, all evaluated by the first author. In every case, surgery was indicated for AKP in patients whose only identifiable pathological finding was excessive ETT. The mean preoperative ETT measured 49.26° ± 8.73° (range, 36–72°). Preoperative knee MRI findings were normal in all patients, and TT–TG distances were within normal limits. All patients had completed an adequate course of conservative treatment without symptomatic improvement, and none had undergone previous knee surgery. Only 30.3% of patients with pathological ETT in the first author’s series underwent rotational osteotomy. The remaining 69.7% did not have symptoms and/or disability severe enough to warrant surgery.

Mean VAS pain scores improved from 8 ± 0.91 (range, 6–10) preoperatively to 0.9 ± 1.37 (range, 0–4) at final follow-up. Similarly, the Kujala score increased from a preoperative mean of 43.15 ± 18.46 (range, 12–76) to a postoperative mean of 93.61 ± 3.12 (range, 90–100). Overall, 90% of patients indicated that they would choose to undergo the procedure again.

The specificity of our series, composed exclusively of young women with AKP, no instability, normal MRI, and isolated excessive ETT, provides a uniquely focused perspective rarely captured in broader published cohorts. By minimizing the confounding factors typically present in heterogeneous populations, these data suggest that patients matching this precise profile may experience particularly favorable outcomes after rotational correction, with improvements at the upper end of those previously reported.

### 3.9. Complications

Intraoperative complications following high tibial rotational osteotomy have been reported with a pooled incidence of 3.8% (95% CI: 2.4–6.0%) [51]. Beyond the general risks inherent to osteotomy procedures—including nonunion, delayed union, infection, loss of correction, fracture, implant fatigue failure, overcorrection or undercorrection, and neurovascular injury, all of which are uncommon—one complication appears to be more specifically associated with this technique: peroneal nerve palsy.

Peroneal nerve injury represents the most frequently reported complication, with an incidence of 1.3%, and most cases consist of transient neurapraxia that resolves spontaneously within a few months [51]. The vulnerability of the nerve is primarily related to increased tension generated during internal rotation of the distal tibial fragment. In addition, reintervention was necessary in a pooled proportion of 13.0% of cases (95% CI: 2.9–27.2%), most commonly for implant removal (n = 158; 28.3%) [51].

In our series, one patient (3.8%) developed a transient peroneal nerve palsy, with full recovery at 9 months. This slightly higher incidence compared with pooled estimates is likely related to the small sample size and the degree of rotational correction required in that particular case.

## 4. Conclusions

Pathological ETT is often overlooked as a cause of AKP, yet it should be systematically assessed during the physical examination of every AKP patient. It is important to emphasize that not all AKP patients with pathological ETT are candidates for surgery. Surgery is indicated only in cases with severe and disabling AKP that is recalcitrant to an adequate conservative treatment. In appropriately selected cases, IRTO represents a reliable treatment option for symptomatic excessive ETT, offering favourable outcomes with minimal complications.

## Data Availability

No new data were created or analyzed in this study.

## References

[1] Collins N.J., Bierma-Zeinstra S.M., Crossley K.M., van Linschoten R.L., Vicenzino B., van Middelkoop M. (2013). Prognostic factors for patellofemoral pain: A multicentre observational analysis. Br. J. Sports Med..

[2] Sanchis-Alfonso V., Teitge R.A. (2022). Torsional abnormality: The forgotten issue in the diagnosis and treatment of the anterior knee pain patient. J. Clin. Med..

[3] Sanchis-Alfonso V., Teitge R.A. (2026). Anterior knee pain and femoral torsion in female patients: Rationale and outcomes of rotational femoral osteotomy. J. Exp. Orthop..

[4] Sanchis-Alfonso V., Ramirez-Fuentes C., Montesinos-Berry E., Domenech-Fernandez J. (2025). High prevalence of femoral and tibial torsional abnormalities in female patients with anterior knee Pain resistant to conservative treatment: A CT-Based study. J. Exp. Orthop..

[5] James S.L., Kennedy J.C. (1979). Chondromalacia of the Patella in the Adolescent. The Injured Adolescent Knee.

[6] Teitge R.A. (2017). Patellofemoral disorders correction of rotational malalignment of the lower extremity. Noyess, Knee Disorders: Surgery, Rehabilitation, Clinical Outcomes.

[7] Teitge R.A. (2012). Does lower limb torsion matter?. Tech. Knee Surg..

[8] Teitge R.A. (2018). The power of transverse plane limb mal- alignment in the genesis of anterior knee pain—Clinical relevance. Ann. Jt..

[9] Meister K., James S.L. (1995). Proximal tibial derotation osteotomy for anterior knee pain in the miserably malaligned extremity. Am. J. Orthop..

[10] Cooke T.D., Price N., Fisher B. (1990). The inwardly pointing knee. An unrecognized problem of external rotational malalignment. Clin. Orthop. Relat..

[11] Sanchis-Alfonso V., Domenech-Fernandez J., Ferras-Tarrago J., Rosello-Añon A., Teitge R.A. (2022). The incidence of complications after derotational femoral and/or tibial osteotomies in patellofemoral disorders in adolescents and active young patients: A systematic review with meta-analysis. Knee Surg. Sports Traumatol. Arthrosc..

[12] Flandry F., Hughston J.C. (1995). Complications of extensor mechanism surgery for patellar malalignment. Am. J. Orthop..

[13] Stevens P.M., Gililland J.M., Anderson L.A., Mickelson J.B., Nielson J., Klatt J.W. (2014). Success of torsional correction surgery after failed surgeries for patellofemoral pain and instability. Strateg. Trauma. Limb Reconstr..

[14] Levens A.S. (1948). Transverse rotation of the segments of the lower extremity in locomotion. J. Bone Jt. Surg..

[15] Sanchis-Alfonso V., Ramírez-Fuentes C., Teitge R.A. (2024). Severe inwardly pointing knee after medial patellofemoral ligament reconstruction: A case report. J. ISAKOS.

[16] Lee T.Q., Morris G., Csintalan R.P. (2003). The influence of tibial and femoral rotation on patellofemoral contact area and pressure. J. Orthop. Sports Phys. Ther..

[17] Lee T.Q., Yang B.Y., Sandusky M.D., McMahon P.J. (2001). The effects of tibial rotation on the patellofemoral joint: Assessment of the changes in in situ strain in the peripatellar retinaculum and the patellofemoral contact pressures and areas. J. Rehabil. Res. Dev..

[18] Snow M. (2021). Tibial torsion and patellofemoral pain and instability in the adult population: Current concept review. Curr. Rev. Musculoskelet. Med..

[19] Barton K.I., Boldt K.R., Sogbein O.A., Steiner N.J., Moatshe G., Arendt E., Getgood A. (2024). Femoral internal torsion greater than twenty-five degrees and/or external tibial torsion greater than thirty degrees as measured by computed tomography are threshold values for axial alignment correction in patellofemoral instability. J. ISAKOS.

[20] Ferreira B., Gomes E., Figueiredo I., Ribeiro R., Valente C., Delgado D., Sánchez M., Andrade R., Espregueira-Mendes J. (2024). Derotational high tibial osteotomy in cases of anterior knee pain and/or patellofemoral instability: A systematic review. J. ISAKOS.

[21] Fouilleron N., Marchetti E., Autissier G., Gougeon F., Migaud H., Girard J. (2010). Proximal tibial derotation osteotomy for torsional tibial deformities generating patello-femoral disorders. Orthop. Traumatol. Surg. Res..

[22] Manilov R., Chahla J., Maldonado S., Altintas B., Manilov M., Zampogna B. (2020). High tibial derotation osteotomy for distal extensor mechanism alignment in patients with squinting patella due to increased external tibial torsion. Knee.

[23] El Attal R., Kaiser P., Dejour D., Zaffagnini S., Arendt E.A., Sillanpaa P. (2019). Derotational osteotomies in patella instability. Patellofemoral Pain, Instability and Arthritis.

[24] Sanchis-Alfonso V., Montesinos-Berry E., Ordoño-Dominguez F. (2025). Infratuberositary derotational tibial osteotomy for anterior knee pain patients in pathological external tibial torsion. Technical note. J. ISAKOS.

[25] Jend H.H., Heller M., Dallek M., Schoettle H. (1981). Measurement of tibial torsion by computer tomography. Acta Radiol. Diagn..

[26] Sanchis-Alfonso V., Ramirez-Fuentes C., Yañez-Rodriguez J., Parra-Calabuig L., Lopez-Vega M., Domenech-Fernandez J. (2025). In females with anterior knee pain, the infratuberositary contribution to external tibial torsion increases with torsion severity and does not correlate with tibial tubercle lateralization. J. Exp. Orthop..

[27] Cameron J.C., Saha S. (1996). External tibial torsion: An underrecognized cause of recurrent patellar dislocation. Clin. Orthop. Relat. Res..

[28] Delgado E.D., Schoenecker P.L., Rich M.M., Capelli A.M. (1996). Treatment of severe torsional malalignment syndrome. J. Pediatr. Orthop..

[29] Dickschas J., Tassika A., Lutter C., Harrer J., Strecker W. (2017). Torsional osteotomies of the tibia in patellofemoral dysbalance. Orthop. Trauma. Surg..

[30] Drexler M., Dwyer T., Dolkart O., Goldstein Y., Steinberg E.L., Chakravertty R. (2014). Tibial rotational osteotomy and distal tuberosity transfer for patella subluxation secondary to excessive external tibial torsion: Surgical technique and clinical outcome. Knee Surg. Sports Traumatol. Arthrosc..

[31] Leonardi F., Rivera F., Zorzan A., Ali S.M. (2014). Bilateral double osteotomy in severe torsional malalignment syndrome: 16 years follow-up. J. Orthop. Traumatol..

[32] Paulos L., Swanson S.C., Stoddard G.J., Barber-Westin S. (2009). Surgical correction of limb malalignment for instability of the patella: A comparison of 2 techniques. Am. J. Sports Med..

[33] Server F., Miralles R.C., Garcia E. (1996). Medial rotational tibial osteotomy for patellar instability secondary to lateral tibial torsion. Int. Orthop..

[34] Elsheikh A.A., Cross G.W.V., Wright J., Goodier D., Calder P. (2023). Miserable malalignment syndrome associated knee pain: A case for infra-tubercle tibial de-rotation osteotomy using an external fixator. J. Orthop. Surg. Res..

[35] Bruce W.D., Stevens P.M. (2004). Surgical correction of miserable malalignment syndrome. J. Pediatr. Orthop..

[36] Van Heerwaarden R.J., Teitge R.A., Parikh S.N. (2020). Rotational osteotomy of the tibia. Patellar Instability.

[37] Kuroda R., Kambic H., Valdevit A., Andrish J.T. (2001). Articular cartilage contact pressure after tibial tuberosity transfer. A cadaveric study. Am. J. Sports Med..

[38] Nakagawa K., Wada Y., Minamide M., Tsuchiya A., Moriya H. (2002). Deterioration of long- term clinical results after the Elmslie-Trillat procedure for dislocation of the patella. J. Bone Jt. Surg. Br..

[39] Balcarek P., Radebold T., Schulz X., Vogel D. (2019). Geometry of torsional malalignment syndrome: Trochlear dysplasia but not torsion predicts lateral patellar instability. Orthop. J. Sports Med..

[40] Diederichs G., Kohlitz T., Kornaropoulos E., Heller M.O., Vollnberg B., Scheffler S. (2013). Magnetic resonance imaging analysis of rotational alignment in patients with patellar dislocations. Am. J. Sports Med..

[41] Kaiser P., Loth F., Attal R., Kummann M., Schuster P., Riechelmann F. (2020). Static patella tilt and axial engagement in knee extension are mainly influenced by knee torsion, the tibial tuber- cle-trochlear groove distance (TTTG), and trochlear dysplasia but not by femoral or tibial torsion. Knee Surg. Sports Traumatol. Arthrosc..

[42] Pace J.L., Drummond M., Brimacombe M., Cheng C., Chiu D., Luczak S.B., Shroff J.B., Zeng F., Kanski G.M., Kakazu R. (2023). Unpacking the tibial tubercle-trochlear groove distance: Evaluation of rotational factors, trochlear groove and tibial tubercle position, and role of trochlear dysplasia. Am. J. Sports Med..

[43] Camp C.L., Heidenreich M.J., Dahm D.L., Stuart M.J., Levy B.A., Krych A.J. (2016). Individualizing the tibial tubercle-trochlear groove distance: Patellar instability ratios that predict recurrent instability. Am. J. Sports Med..

[44] Ackermann J., Hasler J., Graf D.N., Fucentese S.F., Vlachopoulos L. (2022). The effect of native knee rotation on the tibial-tubercle-trochlear-groove distance in patients with patellar instability: An analysis of MRI and CT measurements. Arch. Orthop. Trauma. Surg..

[45] Suomalainen J.S., Regalado G., Joukainen A., Kääriäinen T., Könönen M., Manninen H., Sipola P., Kokki H. (2018). Effects of knee flexion and extension on the tibial tuberosity-trochlear groove (TT-TG) distance in adolescents. J. Exp. Orthop..

[46] Pascual-Leone N., Jahandar A., Davie R., Bram J.T., Chipman D.E., Imhauser C.W., Green D.W. (2024). Femorotibial rotation is linearly associated with tibial tubercle-trochlear groove distance: A cadaveric study. J. ISAKOS.

[47] Smith B.W., Millar E.A., Jones K.C., Elias J.J. (2018). Variations in tibial tuberosity to trochlear groove and posterior cruciate ligament distances due to tibial external and valgus rotations. J. Knee Surg..

[48] Sieberer J.M., Park N., Desroches S., Brennan K., Rancu A., McGinley B., Manafzadeh A.R., Segal N.A., Felson D., Tommasini S.M. (2025). Breaking down Tibial Tuberosity to Trochlear Groove Distance (TT-TG) into two Components to Enable Patient-Specific Treatment Strategies. J. ISAKOS.

[49] Sanchis-Alfonso V., Castellano-Curado J., Gulmini M., Ramirez-Fuentes C., Teitge R.A., Domenech-Fernandez J. (2026). Knee rotation significantly increases measured TT-TG distance in female patients with anterior knee pain: Findings after rotational measurement correction. J. Exp. Orthop..

[50] Tensho K., Akaoka Y., Shimodaira H., Takanashi S., Ikegami S., Kato H., Saito N. (2015). What components comprise the measurement of the tibial tuberosity-trochlear groove distance in a patellar dislocation population?. J. Bone Jt. Surg. Am..

[51] Figueiredo I., Valente C., Ribeiro R., Ferreira B., Gomes E., Delgado D., Sánchez M., Andrade R., Espregueira-Mendes J. (2023). Complications after knee derotational osteotomies in patients with anterior knee pain and/or patellofemoral instability: A systematic review with meta-analysis. Knee Surg. Sports Traumatol. Arthrosc..

